# RETRACTION: Identification of Novel Biomarkers for Childhood‐Onset Systemic Lupus Erythematosus Using Machine Learning Algorithms and Immune Infiltration Analysis

**DOI:** 10.1111/srt.70278

**Published:** 2025-10-21

**RETRACTION**: Y. Deng, and Y. Sun, “Identification of Novel Biomarkers for Childhood‐Onset Systemic Lupus Erythematosus Using Machine Learning Algorithms and Immune Infiltration Analysis,” *Skin Research and Technology* 30, no. 8 (2024): e13880, https://doi.org/10.1111/srt.13880.

The above article, published online on 31 July 2024 in Wiley Online Library (wileyonlinelibrary.com), has been retracted by agreement between *Skin Research and Technology*; and John Wiley & Sons Ltd. The article was accepted for publication following an insufficient peer review process that does not meet the standards of the journal's peer review policy. Furthermore, methodological flaws have been identified, undermining the reliability of the findings. Accordingly, the article has been retracted. The authors have been informed of the retraction decision and disagree with it.

